# Conversion From Twice-Daily Tacrolimus to Once-Daily Extended Release Tacrolimus (LCPT): The Phase III Randomized MELT Trial

**DOI:** 10.1111/ajt.12035

**Published:** 2012-12-21

**Authors:** S Bunnapradist, K Ciechanowski, P West-Thielke, S Mulgaonkar, L Rostaing, B Vasudev, K Budde, on behalf of the MELT investigators

**Affiliations:** a David Geffen School of Medicine at UCLALos Angeles, CA; b Department of Nephrology, Pomeranian Medical UniversitySzczecin, Poland; c University of Illinois Hospital and Health Science SystemChicago, IL; d St. Barnabas Health Care SystemLivingston, NJ; e University Hospital Toulouse-RangueilToulouse, France; f Medical College of WisconsinMilwaukee, WI; g Department of Nephrology, Charité UniversitätsmedizinBerlin, Germany

**Keywords:** Conversion, immunosuppression, kidney transplantation, tacrolimus

## Abstract

Phase III noninferiority trial examining efficacy and safety of converting stable renal transplant recipients from twice-daily tacrolimus to a novel extended-release once-daily tacrolimus formulation (LCPT) with a controlled agglomeration technology. Controls maintained tacrolimus twice daily. The primary efficacy endpoint was proportion of patients with efficacy failures (death, graft failure, locally read biopsy-proven acute rejection [BPAR], or loss to follow-up) within 12 months. Starting LCPT dose was 30% lower (15% for blacks) than preconversion tacrolimus dose; target trough levels were 4–15 ng/mL. A total of 326 patients were randomized; the mITT population (n = 162 each group) was similar demographically in the two groups. Mean daily dose of LCPT was significantly (p < 0.0001) lower than preconversion tacrolimus dose at each visit; mean trough levels between groups were similar. There were four efficacy failures in each group; safety outcomes were similar between groups. Frequency of premature study drug discontinuation was LCPT: 12% versus tacrolimus twice daily: 5% (p = 0.028). LCPT demonstrated noninferiority to tacrolimus twice daily in efficacy failure rates. LCPT may offer a safe and effective alternative for converting patients to a once-daily formulation. Compared to currently available tacrolimus formulation, LCPT requires lower doses to achieve target trough levels.

## Introduction

Tacrolimus capsules (Prograf®, Astellas Pharma US, Inc.) are indicated for the prophylaxis of organ rejection in patients receiving liver, kidney or heart transplants. Tacrolimus twice daily has proven to be highly effective in preventing acute rejection after kidney transplantation 1 and as such is widely used as part of the immunosuppression regimen for kidney transplant recipients. The latest OPTN data indicated that 89.7% of kidney transplant recipients transplanted in 2009 received tacrolimus prior to hospital discharge and that at 1 year posttransplantation, 90.0% of patients (transplanted in 2008) were on tacrolimus 2.

Pharmacodynamic studies have revealed that, depending on the time following transplantation, maintaining whole-blood tacrolimus trough levels between 4 ng/mL and 15 ng/mL provides sufficient protection against acute rejection and limits the occurrence of adverse events (AE) 3,4. The management of tacrolimus blood levels is complicated by variable patient absorption, interaction with food and concomitant medications, and the relatively low bioavailability of tacrolimus from the tacrolimus twice-daily formulation (17 ± 10%) in adult kidney transplant patients 5. Taken together, this may lead to variable drug exposure and high intraindividual variability, which may be associated with inferior outcomes 6. In addition, twice-daily dosing is not optimal as multiple-daily dosing is associated with increased risk for nonadherence 7–9, which can lead to acute rejection 10 and, in serious cases, graft failure 11.

An extended-release formulation of tacrolimus designed for once-daily administration (LCP-Tacro™ tablets [LCPT], Veloxis Pharmaceuticals, Hørsholm, Denmark) has been developed utilizing a proprietary drug delivery technology (MeltDose®, Veloxis Pharmaceuticals, Hørsholm, Denmark), which is designed to improve the bioavailability of drugs with low water solubility. This technology decreases a drug's particle size to become individual molecules, (“solid solution”) the most bioavailable form of the drug. Tacrolimus in LCPT is homogenously embedded in a vehicle system which in turn is homogenously distributed in the tablet matrix. Specifically, the controlled agglomeration of LCPT results in a granulate directly compressed into a tablet. LCPT is designed to deliver the dose throughout the GI tract, providing stable consistent absorption over the full day. Phase II studies of *de novo* and stable renal recipients showed reliable pharmacokinetic (PK) parameters with approximately 30% better bioavailability, only a few treatment failures, and a good safety profile (data on file). AUC_24_ and C_min_ correlation coefficients after 7 and 14 days for *de novo* and converted patients were ≥0.86, demonstrating a robust correlation between LCPT tacrolimus exposure and trough levels. The primary objective of this study was to evaluate the efficacy and safety of LCPT tablets when used to replace tacrolimus twice-daily capsules for maintenance immunosuppression in adult renal transplant patients.

## Methods

The methods are briefly summarized below with a complete description available as Supporting files available online.

### Study design and conduct, patient population and study drug dosing

**Figure 1:**
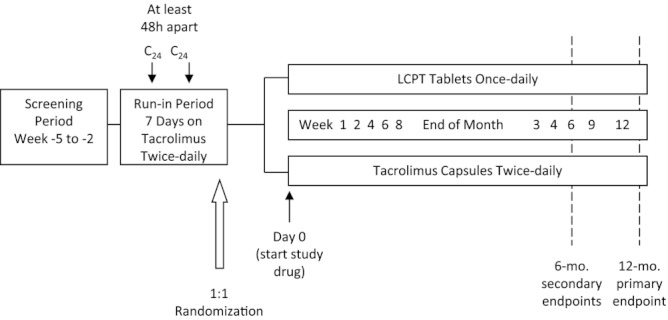
Study design.

This was a two-armed, parallel group, prospective, randomized, open-label, multicenter, phase III, controlled, noninferiority trial (Multicenter Evaluation of LCPT Tablets [MELT trial]; http://ClinicalTrials.gov: NCT00817206) (Figure 1). Institutional Review Board approval was obtained at each participating center, and informed consent was obtained from all patients. The study was undertaken in accordance with the ICH Harmonized Tripartite Guidelines for Good Clinical Practice and conformed to the Declaration of Helsinki.

The study took place between December 23, 2008 and February 7, 2011 at 47 sites (n = 33 US, n = 14 Europe). Stable adult (≥18 years) male and female recipients of a living or deceased donor kidney transplant between 3 months and 5 years before screening, on a stable tacrolimus dose with tacrolimus trough levels within 4–15 ng/mL, who met inclusion criteria were randomly assigned to be converted from tacrolimus twice daily to LCPT or to remain on maintenance therapy with tacrolimus twice daily. Initial dosing of LCPT was 0.7 times the total daily dose of tacrolimus twice daily being taken by the patient before conversion, due to higher bioavailability 12,13. Because black patients require higher doses of tacrolimus to achieve comparable blood concentrations to nonblacks’ 14, and based on preliminary data with LCPT (data on file), black patients were converted using a 0.85 conversion multiplier. All subsequent study drug dose adjustments were based on clinical assessment of the patient and maintenance of target tacrolimus whole blood trough levels within the predefined range of 4–15 ng/mL.

### Study endpoints

The primary efficacy endpoint was proportion of patients with efficacy failures (death, graft failure, locally read BPAR (Banff grade ≥1A), or lost to follow-up) within 12 months in the mITT population (i.e. all patients who received ≥1 dose of study drug).

Secondary efficacy endpoints included efficacy endpoints assessed after 6 months included; incidence of efficacy failure in the per protocol (PP; all ITT patients who completed the study without any major protocol deviations) set; incidence of individual components of the composite efficacy endpoint; incidence of steroid-resistant acute rejection and clinically suspected and treated rejection episodes, severity grades of the first episode of BPAR (Banff grade) and incidence of premature discontinuation of randomly assigned study drug.

The primary safety assessment was the differences between treatment groups at month 12 in the incidence of AEs and the incidence of predefined potentially clinically significant laboratory measures (fasting plasma glucose ≥200 mg/dL; platelet count <100 × 109 cells/L; white blood cell count <2.0 × 10^9^ cells/L; aminotransferases ≥100 U/L; total cholesterol ≥300 mg/dL; low-density lipoprotein cholesterol ≥200 mg/dL; triglycerides ≥500 mg/dL and eGFR <30 mL/min.

Secondary safety endpoints included: the mean change from baseline in estimated creatinine clearance (Cockcroft-Gault) and eGFR; incidence of any opportunistic infection or malignancy within 12 months; the proportion of patients with hemoglobin A1c (HbA1c) ≥6.5%; change from baseline in HbA1c; change from baseline in protein:creatinine ratio; mean daily dose of study drug and whole blood trough tacrolimus level at each study visit and incidence of new onset diabetes (NODM) within 6 months and 12 months (*post hoc* analysis utilizing 2010 ADA criteria [15]).

### Statistical analyses

The noninferiority evaluation for the primary efficacy endpoint was based on the two-sided 95% CIs for the difference (LCPT–tacrolimus twice daily) in the efficacy failure rates between the treatment groups. If the upper bound of the 95% CI for the difference in efficacy failure rates was less than 9%, then LCPT was to be considered noninferior to tacrolimus twice daily in efficacy failure rate. At each visit, descriptive statistics were summarized by the treatment groups. The Fisher exact test was used to compare categorical data between the groups and one-way analysis of variance was used to compare continuous data between the groups. The Paired sample t-test was used to compare change from baseline within a group. *Post hoc* by race (black vs. nonblack subjects) analysis was performed on efficacy parameters and reported adverse events.

## Results

### Patient disposition and baseline characteristics

**Figure 2:**
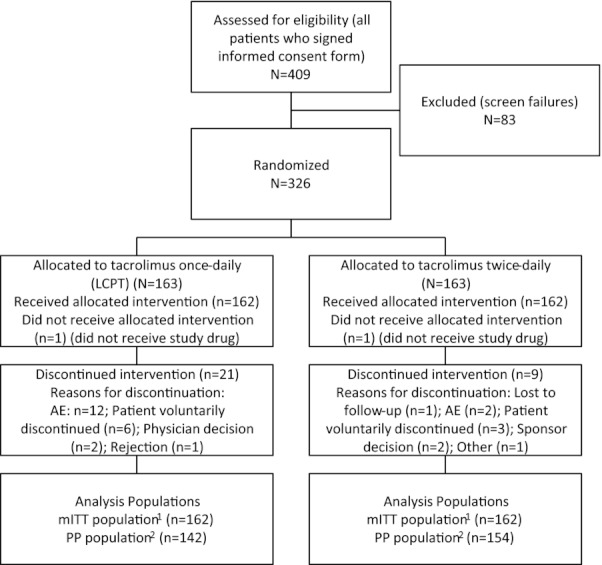
Patient disposition.

A total of 409 patients were enrolled into the study, of which 83 were screen failures and not randomly assigned to study treatment. Overall, 326 patients were randomly assigned to the study, 163 patients in each treatment group, and 296 patients completed the 12-month treatment period (LCPT, n = 142; tacrolimus twice daily, n = 154). One patient in each treatment group was randomly assigned and not dosed; thus, a total of 324 patients (99.4%) were included in the mITT set, 162 in each treatment group (Figure 2).

Demographic characteristics were similar across both treatment groups. The patient population was predominately white (72.7%) and male (67.2%); mean age was 50.3 years. Blacks comprised 21% of the randomized sample (n = 69) (Table 1).

**Table 1:** Patient demographics and baseline characteristics—mITT set

	LCPT N = 162	Tacrolimus twice daily N = 162	p-Value
Age (years), mean (SD)	50.4 (11.75)	50.3 (13.46)	0.965
Sex			0.098
Male	116 (71.6%)	101 (62.3%)	
Female	46 (28.4%)	61 (37.7)	
Race			0.836
White	120 (74.1%)	116 (71.6%)	
Black	35 (21.6%)	34 (21.0%)	
Asian	3 (1.9%)	3 (1.9%)	
Other	4 (2.5%)	9 (5.5%)	
Previous rejection for the current graft, n (%)	20 (12.3%)	23 (14.2%)	0.744
Donor type			0.244
Living	62 (38.3%)	51 (31.5%)	
Deceased	100 (61.7%)	111 (68.5%)	
Number of HLA mismatches			0.539
0	15 (9.3%)	15 (9.3%)	
1	10 (6.2%)	8 (4.9%)	
2	18 (11.1%)	11 (6.8%)	
≥3	119 (73.5%)	128 (79.0%)	
Subjects who had a previous transplant other than the current transplant, n (%)	22 (13.6%)	19 (11.7%)	0.739
Pretransplant diabetes, n (%)	63 (38.9%)	59 (36.4%)	0.647
PRA (%), mean (SD)	10.4 (24.09)	8.9 (19.47)	0.567
Preconversion Prograf total daily dose (mg)	6.1 (3.90)	5.3 (3.35)	0.063
PRA (%), median	0	0	
PRA <5%, n (%)	106 (65.4%)	100 (61.7%)	
Months from transplant to enrollment, mean (SD)	25.9 (16.7)	22.1 (15.2)	0.034

HLA = human leukocyte antigen; PRA = panel-reactive antibody.

### Immunosuppression

**Figure 3:**
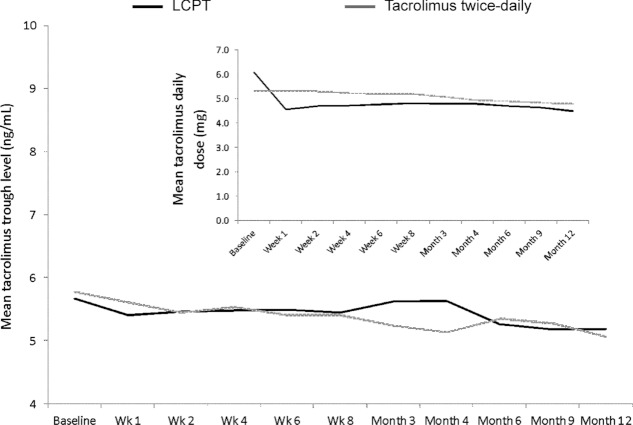
Mean tacrolimus trough levels and mean tacrolimus daily dose during study.

Mean (SE) tacrolimus daily dose at baseline prior to conversion was 6.09 ± 0.31 mg in the LCPT group and 5.34 ± 0.26 mg in the tacrolimus twice-daily group (*p* = 0.06). On average, the reductions in total daily dose were greater in the LCPT group than the Prograf group. Within groups, the reductions in total daily dose (both the mean and percent change from baseline) were highly significant in the LCPT group, whereas the Prograf group showed only slight reductions. The mean daily dose over the 12-month study period was approximately 20% lower compared to baseline for LCPT and fell approximately 3.6% in the tacrolimus twice-daily group. For week 1 the mean (SE) daily dose was 4.6 (0.23) for LCPT and 5.3 (0.26) for tacrolimus twice daily while mean (SD) tacrolimus trough levels were 5.41 (2.43) and 5.61 (1.83) respectively. At each study visit, the mean daily dose of LCPT was significantly lower than preconversion tacrolimus dose (p < 0.0001) (Table 2). Mean tacrolimus trough levels were similar between the groups throughout the study and were within the target range (Table 2 and Figure 3). Immediately postconversion week 1 (days 1–9), 17% of LCPT patients and 13% of tacrolimus twice-daily patients had at least one dose adjustment. Of those, 15% (LCPT) versus 9% (tacrolimus twice daily) of adjustments were done based on trough levels ≥25% outside of the preconversion trough.

**Table 2:** Mean tacrolimus daily dose (mg) and tacrolimus trough levels (ng/mL)

	Mean (SE) tacrolimus daily dose (mg)				Mean (SD) tacrolimus trough level (ng/mL)	
		
	LCPT	p- Value^1^	Tacrolimus twice daily	p- Value^1^	LCPT	Tacrolimus twice daily
Baseline	6.1 (0.31)		5.3 (0.26)		5.67 (2.24)	5.78 (1.94)
Week 1	4.6 (0.23)	<0.0001	5.3 (0.26)	0.7120	5.41 (2.43)	5.61 (1.83)
Week 2	4.7 (0.24)	<0.0001	5.3 (0.26)	0.7338	5.46 (1.99)	5.45 (1.72)
Week 4	4.7 (0.24)	<0.0001	5.2 (0.26)	0.2967	5.48 (1.98)	5.54 (1.48)
Week 6	4.8 (0.25)	<0.0001	5.2 (0.25)	0.1651	5.49 (1.85)	5.41 (1.48)
Week 8	4.8 (0.25)	<0.0001	5.2 (0.25)	0.1407	5.45 (1.77)	5.41 (1.63)
Month 3	4.8 (0.26)	<0.0001	5.1 (0.25)	0.0228	5.63 (1.94)	5.24 (1.41)
Month 4	4.8 (0.27)	<0.0001	4.9 (0.24)	0.0003	5.64 (1.90)	5.14 (1.40)
Month 6	4.7 (0.27)	<0.0001	4.9 (0.24)	<0.0001	5.27 (1.80)	5.35 (1.53)
Month 9	4.7 (0.27)	<0.0001	4.8 (0.24)	<0.0001	5.18 (1.56)	5.28 (1.80)
Month 12	4.5 (0.26)	<0.0001	4.8 (0.25)	<0.0001	5.19 (1.99)	5.07 (1.30)
Overall TDD	4.7 (0.25)		4.9 (0.23)			
Overall CFB (%)	−19.6 (2.74)		−3.6 (1.92)			

1 Paired sample *t*-test for change from baseline.

TDD = total-daily dose; CFB = change from baseline.

The proportion of patients requiring at least one dose adjustment was LCPT, 80.2%, and tacrolimus twice daily, 67.9%; the mean (SE) number of dose adjustments per patient was LCPT, 2.0 (0.15), and tacrolimus twice daily, 1.7 (0.14).

Mean (SD) daily dose for blacks was greater than that for nonblacks; among blacks the mean daily dose was similar between the LCPT (blacks: 7.6 mg [3.3]; nonblacks: 3.9 mg [2.5]) and tacrolimus twice daily (blacks: 7.3 mg [3.9]; nonblacks: 4.5 mg [2.5]) groups. Mean tacrolimus trough levels were similar between groups and within the target throughout the study.

### Primary efficacy endpoint

The primary efficacy failure rate was 2.5% (n = 4) for both the LCPT and tacrolimus twice-daily groups; the 95% CI for the treatment difference was −4.21%, 4.21%; the upper bound was well below the prespecified 9.0% noninferiority margin.

For centrally read biopsies, the efficacy failure rate was lower for LCPT (n = 3, 1.9%) than for tacrolimus twice daily (n = 6, 3.7%), and the treatment difference (95% CI) was −1.85% (−6.51%, 2.30%), which was not significant (Table 3).

**Table 3:** Primary efficacy results, LCPT vs. tacrolimus twice daily—mITT set

	Local pathology reading—primary efficacy endpoint	
	
	LCPT* (n = 162)	Tacrolimus twice daily* (n = 162)
		
Efficacy failure, n (%)	4 (2.5)	4 (2.5)
Treatment difference (95% CI)	0% (−4.2,+4.2)	
p-Value	>0.999	
Individual efficacy components		
Death, n (%)	2 (1.2)	1 (0.6)
Graft loss, n (%)	0 (0.0)	0 (0.0)
Lost to f/u, n (%)	0 (0.0)	1 (0.6)
BPAR, n (%)	2 (1.2)	2 (1.2)

	Central pathology reading	
	
	LCPT*	Tacrolimus twice daily
BPAR, n (%)	1 (0.6)	4 (2.5)
p-Value	0.371	

* Modified intent-to-treat (mITT): received one dose of study drug; the prespecified noninferiority margin was 9%.

### Efficacy endpoints among blacks (post hoc analysis)

Among blacks the overall efficacy failure rate based on locally read biopsies was lower for LCPT (0%) than for tacrolimus twice daily (n = 2, 5.88%; treatment difference [95% CI]: −5.88% [−13.79, 2.03%]). By central reading, efficacy failure was LCPT: n = 0 versus tacrolimus twice daily n = 3 (treatment difference [95% CI]: −8.82% [−18.36, 0.71%]).

### Secondary efficacy endpoints

Frequency of premature discontinuation of study drug within 12 months, though significantly (p = 0.028) greater in the LCPT (n = 20 patients) versus tacrolimus twice-daily (n = 8) group, was low in both groups. Incidences of efficacy failure, graft loss or death or acute rejection within 6 months were not frequent and were similar between the drug groups. Efficacy failure within 12 months in the PP set was similarly infrequent in both groups (LCPT, n = 1; tacrolimus twice daily, n = 2).

Patient survival at 12 months was 98.8% and 99.4% for the LCPT and tacrolimus twice-daily treatment groups, respectively, and no statistically significant difference was observed between the two treatments. Death censored graft survival at 12 months was 100.0% for both treatments.

### Safety

The incidence of AEs was similar between the LCPT and tacrolimus twice-daily treatment groups. Overall, 268 patients (82.7%) experienced 1270 treatment-emergent AEs, 135 (83.3%) in the LCPT group and 133 (81.6%) in the tacrolimus twice-daily group (Table 4). The most frequently reported treatment-emergent AEs included diarrhea, urinary tract infection (UTI), increased blood creatinine, nasopharyngitis, headache, upper respiratory tract infection, peripheral edema and hypertension. All of these events occurred in both treatment groups (Table 5).

**Table 4:** Primary and secondary safety results, LCPT versus tacrolimus twice daily

	LCPT N = 162	Tacrolimus twice daily N = 162	p- Value
Primary safety endpoints			
Number (%) of patients with TEAEs^1^	135 (83.3%)	133 (82.1%)	
incidence of predefined potentially clinically significant laboratory measures			
Fasting plasma glucose ≥200 mg/dL, new-onset/atrisk (%)	19/156 (12.2%)	10/149 (6.7%)	0.120
Platelet count <100 × 109 cells/L	0/161 (0.0%)	0/162 (0.0%)	N/A
White blood cell (WBC) count <2.0 × 109 cells/L	1/161 (0.6%)	1/162 (0.6%)	>0.999
Aminotransferases ≥100 U/L	2/160 (1.25%)	1/162 (0.6%)	0.621
Total cholesterol ≥300 mg/dL	4/161 (2.5%)	1/162 (0.6%)	0.214
Low-density lipoprotein (LDL) cholesterol ≥200 mg/dL	2/162 (1.2%)	1/162 (0.6%)	>0.999
Triglycerides ≥500 mg/dL	2/160 (1.25%)	0/161 (0.0%)	0.248
eGFR <30 mL/min	5/162 (3.1%)	5/159 (3.1%)	>0.999
Secondary safety endpoints			
Incidence of NODM within 6 months, new-onset/at-risk (%)	7/90 (7.78%)	8/95 (8.42%)	1.000
Incidence of NODM within 12 months, new-onset/at-risk (%)	9/90 (10.00%)	10/95 (10.53%)	1.000
Incidence of opportunistic infection within 12 months, n (%)	9 (5.6%)	10 (6.2%)	>0.999
Incidence of malignancy within 12 months, n (%)	8 (4.9%)	9 (5.6%)	>0.999

All analyses based on the mITT set; NODM: new-onset diabetes mellitus (fasting plasma glucose ≥126 mg/dL, HbA1c >6.5% at least 3 months after randomization, insulin requirement for ≥30 days, or need for an oral hypoglycemic agent in patients with no prior medical history consistent with diabetes); TEAE: treatment emergent adverse event.

1 Any AE that started after the first dose and within 30 days of the final dose of study drug; at-risk patients are the patients whose laboratory values at baseline do not meet the predefined abnormal criteria.

**Table 5:** Summary of adverse events, LCPT versus tacrolimus twice daily

	LCPT N = 162	Tacrolimus twice daily N = 162
Number of TEAEs	699	571
Number of TEAEs suspected of being related to study drug	50 (7.1%)	32 (5.6%)
Number of patients with at least 1 TEAE	135 (83.3%)	133 (82.1%)
TEAEs occurring in ≥5% of patients		
Diarrhea	22 (13.6%)	15 (9.3%)
UTI	14 (8.6%)	22 (13.6%)
Blood creatinine increased	20 (12.3%)	14 (8.6%)
Nasopharyngitis	15 (9.3%)	18 (11.1%)
Headache	15 (9.3%)	11 (6.8%)
Upper respiratory infection	12 (7.4%)	14 (8.6%)
Edema peripheral	11 (6.8%)	10 (6.2%)
Hypertension	7 (4.3%)	10 (6.2%)
Number of serious TEAEs (STEAE)	52	42
Number of STEAEs suspected of being related to study drug	4 (2.5%)	4 (2.5%)
Number of patients with at least one STEAE	36 (22.2%)	26 (16.0%)

Treatment emergent is defined as any AE that started after first dose and within 30 days of the final dose of study drug.

Serious AEs (SAE) were reported for 36 patients (22.2%) in the LCPT group, and 26 patients (16.0%) in the tacrolimus twice-daily group. During the study, two patients in the LCPT group died of cardiac arrest. Neither of these deaths was suspected to be related to study drug. Additionally, two patients died during the follow-up period, both of whom were discontinued from the study due to AEs: one patient in the LCPT group died of cardiac arrest approximately 4 months after discontinuing the study (outside of 12-month reporting period) and one patient in the tacrolimus twice-daily group died of lung cancer approximately 7 months after discontinuing the study (within the 12-month reporting period). The most frequently reported SAE was UTI (LCPT, n = 3, 1.9%; tacrolimus twice daily, n = 4, 2.5%). Most SAEs were not suspected to be related to study drug. Most patients with SAEs had tacrolimus trough levels within the therapeutic range.

**Figure 4:**
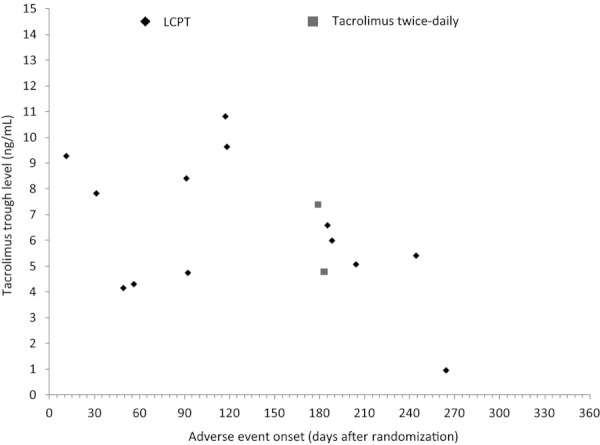
Tacrolimus trough levels preceding adverse event leading to discontinuation.

Fifteen AEs in 15 patients (4.6%) led to discontinuation of study treatment, 13 events in 13 patients (8.0%) in the LCPT group and two events in 2 patients (1.2%) in the tacrolimus twice-daily group. Four of the events leading to discontinuation that occurred in patients in the LCPT group were suspected to be related to study drug as follows: grade 2 drug level fluctuating, grade 3 abnormal renal function test, grade 2 pain in extremity and grade 1 toxic nephropathy. Almost all patients who discontinued study drug due to an AE had tacrolimus trough levels within the protocol specified normal range (Figure 4). The AEs leading to discontinuation did not show any trend in terms of type of event or timing of event. Most patients experienced treatment-emergent AEs that were mild (75.9% overall, 76.5% and 75.3% in the LCPT and tacrolimus twice-daily groups, respectively) or moderate (42.0% overall, 43.2% and 40.7% in the LCPT and tacrolimus twice-daily groups, respectively) in severity. Thirty-one events in 19 patients (11.7%) in the LCPT group and 20 events in 9 patients (5.6%) in the tacrolimus twice-daily group were considered severe. The majority of the patients experienced events that were not suspected to be related to study drug (Table 5).

**Figure 5:**
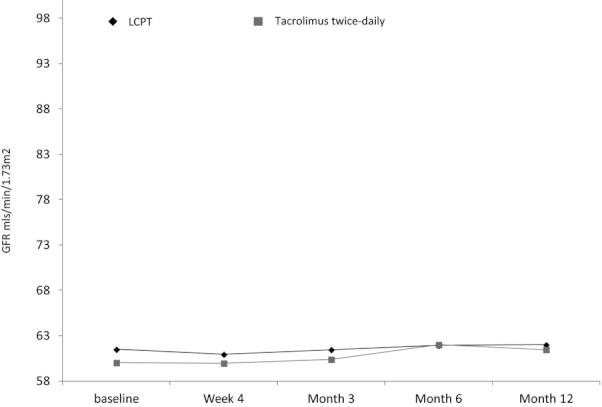
Mean GFR during the study.

As displayed in Table 4, there were no significant differences between the LCPT and tacrolimus twice-daily groups in predefined potentially clinically significant laboratory measures, incidence of NODM, opportunistic infection or malignancies. Mean change from baseline in estimated creatinine clearance and GFR was minimal for both LCPT and tacrolimus twice daily, and the results were similar for both treatments (Figure 5). Among blacks, 88.6% (n = 31) of patients in the LCPT group and 97.1% (n = 33) in the tacrolimus twice-daily group experienced at least one treatment-emergent AE; 28.6% (n = 10) of black patients in the LCPT group and 20.6% (n = 7) in the tacrolimus twice-daily group experienced at least one SAE.

## Discussion

The results reported here are from the first phase III trial to examine the efficacy and safety of converting stable kidney transplant recipients to LCPT from tacrolimus twice daily. This open-label, randomized study in 324 stable kidney transplant recipients demonstrated that patients can be successfully converted from tacrolimus twice daily to LCPT, while maintaining efficacy and safety. By local pathology and central reading, the rates of efficacy failure were equivalent between the groups.

This was a noninferiority study focused on a composite efficacy failure endpoint. LCPT provided excellent rejection prophylaxis in a once-daily formulation. Data from phase II studies have shown that LCPT is associated with an increase in bioavailability 12,13. The increased bioavailability has the potential to result in lower drug dosages needed to achieve equivalent therapeutic drug levels. As expected, the required total daily LCPT dose was about 20% lower than preconversion tacrolimus twice-daily dose, while drug levels were stable. Thus, the conversion factor of 0.7 and 0.85 for blacks was adequate to counteract the expected higher bioavailability of LCPT, and the conversion process was safe. Throughout the study, the mean daily LCPT dose was constantly lower than the dose of tacrolimus twice daily administered, while drug levels were similar and within target range. Again, this observation is consistent with LCPT having a consistently greater bioavailability of tacrolimus. In addition to the potential for a lower tacrolimus dose with LCPT, LCPT tablets have an advantageous once-a-day dosing. Multiple daily dosing can contribute to lack of adherence 7–9,16, and posttransplant drug regimens are often associated with high pill burden. Importantly, lack of adherence is common in transplant recipients 17–19, and a recently published paper reported nonadherence to be a major contributor to graft failure 20 and one of the barriers to improving long-term kidney transplant outcomes.

In this study we found that safety outcomes were similar between the groups and consistent with that expected in a kidney transplant population. There was a greater frequency of premature discontinuations in the LCPT group (12%) versus the tacrolimus twice-daily group (5%). However, the frequency of premature discontinuations from LCPT was low compared to that which has been reported from other conversion trials that included maintenance renal transplant recipients 21,22. Regarding the difference in drop-out rates between LCPT and tacrolimus twice daily, it is worthwhile to consider the trial design. All patients in the MELT trial were on and tolerating a stable dose of tacrolimus twice daily prior to randomization into the trial. Furthermore, tacrolimus twice-daily represents a component of the “gold standard” immunosuppressive regimen in kidney transplant with 90% of all kidney transplant recipients in the United States treated with tacrolimus at the time of discharge from the hospital. The MELT study was open-label; patients on LCPT and experiencing an AE could be switched to tacrolimus. Tacrolimus twice-daily treated patients experiencing an AE may not have had an acceptable alternative regimen. It is therefore possible that the threshold for discontinuing tacrolimus twice-daily patients from the study was higher than the threshold for discontinuing LCPT patients. This, in the absence of any pattern in terms of type of event, tacrolimus trough levels, or timing of the events may act to mitigate concerns regarding the numeric imbalance in these events, and any new drug will likely have higher discontinuation rates. There were a total of four deaths, two of which were not treatment-emergent events. No deaths were considered related to study drug; three were cardiac deaths that occurred in LCPT patients with preexisting cardiac disease and one was due to cancer in a tacrolimus twice-daily treated patient. LCPT successfully demonstrated noninferiority to tacrolimus twice daily in efficacy failure rates in this study with lower doses (∼20% less) of LCPT compared to tacrolimus twice daily. The incidence of AEs, renal function, and the results of other safety evaluations were similar between the two treatment groups in this patient population, and no unexpected toxicity was observed. The results of this study also demonstrate that stable black kidney transplant recipients, a transplant patient population at increased risk of treatment failures, can be safely converted from tacrolimus twice daily to LCPT.

The novel drug delivery technology that improves bioavailability, together with extended drug release has resulted in a novel once-daily dosing version of tacrolimus. The results presented here confirm the higher bioavailability along with the previously reported differences in PK parameters. Our study provides evidence for a safe conversion from tacrolimus twice daily to the novel once-daily dosing regimen and suggests that LCPT is a safe and effective alternative to currently available tacrolimus in kidney transplantation. In addition, a once-daily tacrolimus dosing regimen could improve patient adherence. A large international phase III study is currently ongoing to assess safety, efficacy and PK of LCPT in the *de novo* kidney transplant setting.

## Appendix

MELT investigators and study site affiliations: Rita Alloway, Pharm.D, University of Cincinnati and The Christ Hospital, Cincinnati, OH, USA; Rajendra Baliga, MD, LifeLink Healthcare Institute, Tampa, FL, USA; Kenneth Bodziak, MD, University Hospital Cleveland, Cleveland, OH, USA; Daniel Brennan, MD, Washington University at St. Louis, St. Louis, MO, USA; Suphamai Bunnapradist, MD, David Geffen School of Medicine at UCLA, USA; Laurence Chan, MD, University of Colorado, Denver, CO, USA; Javier Chapochnick-Friedmann, MD, Montefiore Medical Center, Bronx, NY, USA; Gaetano Ciancio, MD, University of Miami, Miami, FL, USA; David Cohen, MD, NY-Presbyterian Hospital (Columbia), New York, NY, USA; Laskow David, MD, Robert Wood Johnson Medical School, New Brunswick, NJ, USA; Standon Dodson, MD, Rush University Medical Center, Chicago, IL, USA; Robert Gaston, MD, University of Alabama Birmingham, Birmingham, AL, USA; Reginald Gohh, MD, Rhode Island Hospital, Providence, RI, USA; Devon John, MD, New York University Medical Center, New York, NY, USA; Anne King, MD, Virginia Commonwealth University, Richmond, VA, USA; Richard Knight, MD, Methodist Hospital, Houston, TX, USA; Sanjay Kulkarni, MD, Yale New Haven Hospital, New Haven, CT, USA; Paul Kuo, MD, Duke University Medical Center, Durham, NC, USA; Jun Lee, MD, Rogosin Institute (NY-Presbyterian Hospital/Cornell), New York, NY, USA; Herwig-Ulf, Meier-Kriesche, MD, University of Florida, Gainesville, FL, USA; Shamkant Mulgaonkar, MD, St. Barnabas Health Care System, Livingston, NJ, USA; Kumar Mysore, MD, Drexel University, Philadelphia, PA, USA; Bobby Nibhanupudy, MD, Florida Hospital Medical Center, Orlando, FL, USA; Charles Nolan, MD, University of Texas San Antonio, San Antonio, TX, USA; Oleh Pankewycz, MD, Buffalo General Hospital, Buffalo, NY, USA; Martha Pavlakis, MD, Beth Israel Deaconess Med Center, Boston, MA, USA; John Pirsch, MD, University of Wisconsin, Madison, WI, USA; Steven Steinberg, MD, F.A.C.P., F.A.C.N., California Institute of Renal Research, CIRR, San Diego, CA, USA; Karin True, MD, University of North Carolina, Chapel Hill, NC, USA; Brahm Vasudev, MD, Froedtert Memorial Lutheran Hospital, Milwaukee, WI, USA; Patricia West-Thielke, Pharm.D, University of Illinois Hospital and Health Science System, Chicago, IL, USA; Carlos Zayas, MD, Piedmont Hospital, Atlanta, GA, USA; Diego Cantarovich, MD, PhD, Hôtel Dieu-Univ Nantes, Nantes, France; Georges Mourad, MD, PhD, Hôpital Lapeyronie, Montpellier, France; Lionel Rostaing, MD, PhD, University Hospital Toulouse-Rangueil, France; Klemens Budde, MD, Department of Nephrology, Charité Universitätsmedizin Berlin, Berlin, Germany; Jens Lutz, MD, Klinikum rechts der Isar der Technische Universität München, München, Germany; Kazimierz Ciechanowski, MD, PhD, Department of Nephrology, Pomeranian Medical University, Szczecin, Poland; Michal Ciszek, Instytut Transplantologii AM, Szpital Kliniczny Dzieciątka Jezus, Warsaw, Poland; Michal Nowicki, SPZOZ Uniwersytecki Szpital Kliniczny nr 1 im. Norberta Barlickiego Uniwersytetu Medycznego w Lodzi, Lodz, Poland; Josep Maria Grinyó Boira, MD, PhD, Hospital Universitari De Bellvitge, Barcelona, Spain; Juan Carlos Ruiz San Millan, MD, University Hospital Marques de Valdecilla, Santander, Spain; Carme Facundomolas, MD, Fundacion Puigvert, Barcelona, Spain; Alan Jardine, MD, Western Infirmary, Glasgow, UK; Richard Baker, MD, St. James's University Hospital, Leeds, UK; Richard Moore, MD, University Hospital of Wales, Cardiff, UK.

Central reader: Parmjeet Randhawa, MD, University of Pittsburgh Medical Center. Kristin Kistler, PhD, from United BioSource Corporation provided medical writing support.

## Abbreviations

AE: adverse event
ADA: American Diabetes Association
BPAR: biopsy-proven acute rejection
CI: confidence interval
eGFR: estimated glomerular filtration rate
FDA: Food and Drug Administration
HLA: human leukocyte antigen
ITT: intent-to-treat
LCPT: LCP-Tacro
MELT: Multicenter Evaluation of LCP-Tacro Tablets
mITT: modified intent-to-treat
NODM: new onset diabetes
OPTN: Organ Procurement and Transplantation Network
PRA: panel-reactive antibody
PP: per protocol
PK: pharmacokinetic
SAE: serious adverse event
TEAE: treatment emergent adverse event
UTI: urinary tract infection

## Supporting Information

**Study Design and Conduct**

**Patient Population**

**Study Drug Dosing**

**Study Endpoints**

**Statistical Analyses**
