# Supplementary material for: Conversion From Twice-Daily Tacrolimus to Once-Daily Extended Release Tacrolimus (LCPT): The Phase III Randomized MELT Trial
Source: Am J Transplant. 2012 Dec 21;13(3):760–9. doi: 10.1111/ajt.12035 (PMC3613750; doi:10.1111/ajt.12035)
Supplement: Supplementary file 1 [file ajt0013-0760-SD1.docx]

**Study Design and Conduct**

This was a 2-armed, parallel group, prospective, randomized, open-label, multicenter, Phase III, controlled, noninferiority trial (Multicenter Evaluation of LCP-Tacro Tablets [MELT-trial]; ClinicalTrials.gov: NCT00817206). Stable kidney transplant recipients were randomly assigned (fixed block randomization scheme) to be converted from tacrolimus twice-daily to LCPT or to remain on maintenance therapy with tacrolimus twice-daily (**Figure 1**).

The study took place between December 23, 2008 and February 7, 2011 at 47 sites (n=33 US, n=14 Europe). Sequential patient numbers were assigned by clinic staff. Institutional Review Board approval was obtained at each participating center, and informed consent was obtained from all patients. The study was undertaken in accordance with the ICH Harmonized Tripartite Guidelines for Good Clinical Practice and conformed to the Declaration of Helsinki.

**Patient Population**

Eligible patients were stable adult (≥18 years) male and female recipients of a living or deceased donor kidney transplant between 3 months and 5 years before screening, on a stable dose (unchanged for ≥30 days, ≥2 mg tacrolimus twice-daily) with tacrolimus trough levels within 4–15 ng/mL. Major exclusion criteria included: recipients of another organ or a bone marrow transplant; administration of sirolimus or everolimus within 3 months before screening; mycophenolate doses (Cellcept® or Myfortic®) that were not stable for at least 4 weeks before screening; acute rejection requiring antibody therapy within 3 months before screening; and patients with an estimated glomerular filtration rate (eGFR; MDRD7) of <30 mL/min at screening.

**Study Drug Dosing**

Initial dosing of LCPT was 0.7 times the total daily dose of tacrolimus twice-daily being taken by the patient before conversion, due to higher bioavailability.([12](#_ENREF_12), [13](#_ENREF_13)) Because black patients require higher doses of tacrolimus to achieve comparable blood concentrations to whites’,([14](#_ENREF_14)) and based on preliminary data with LCPT (data on file), black patients were converted using a 0.85 conversion multiplier. All subsequent study drug dose adjustments were based on clinical assessment of the patient and maintenance of target tacrolimus whole blood trough levels within the predefined range of 4–15 ng/mL. The bioanalytical validated methods for assessing tacrolimus levels in human whole blood was conducted using high performance liquid chromatography and tandem mass spectrometry.

**Study Endpoints**

***Primary Efficacy***

The primary efficacy endpoint was proportion of patients with efficacy failures within 12 months, a composite endpoint including events of death, graft failure (return to dialysis for >30 days, allograft nephrectomy, or retransplantation), locally-read BPAR (Banff grade ≥1A), or lost to follow-up in the mITT population (i.e., all patients who received ≥1 dose of study drug).

***Secondary Efficacy***

Secondary efficacy endpoints assessed after 6 and 12 months included: the incidence of efficacy failure; incidence of efficacy failure in the per protocol (PP; all ITT patients who completed the study without any major protocol deviations) set; incidence of death or graft failure; incidence of BPAR; incidence of steroid-resistant acute rejection (assessed by the need for antibody therapy after course of corticosteroids); proportion of severity grades of the first episode of BPAR (Banff grade); incidence of clinically suspected and treated rejection episodes (treated acute rejection without confirmatory evidence on a biopsy); and incidence of premature discontinuation of randomly assigned study drug.

***Primary Safety***

The primary safety assessment was the differences between treatment groups at month 12 in the incidence of AEs and the incidence of predefined potentially clinically significant laboratory measures (fasting plasma glucose ≥200 mg/dL; platelet count <100 × 109 cells/L; white blood cell count <2.0 × 10^9^ cells/L; aminotransferases ≥100 U/L; total cholesterol ≥300 mg/dL; low-density lipoprotein cholesterol ≥200 mg/dL; triglycerides ≥500 mg/dL; and eGFR <30 mL/min.

***Secondary Safety***

Secondary safety endpoints included: the mean change from baseline in estimated creatinine clearance (Cockcroft-Gault) and eGFR; incidence of any opportunistic infection or malignancy within 12 months; the proportion of patients with hemoglobin A1c (HbA1c) ≥6.5%; change from baseline in HbA1c; change from baseline in protein:creatinine ratio; mean daily dose of study drug and whole blood trough tacrolimus level at each study visit; and incidence of new onset diabetes (NODM) within 6 months and 12 months (post hoc analysis utilizing 2010 ADA criteria([15](#_ENREF_15))).

**Statistical Analyses**

Based on an expected efficacy failure rate of 6% at 1 year in stable kidney transplant recipients, a 10% drop-out rate, and using a noninferiority margin of 9%, a sample size of 302 patients overall (151 per group) was required to give 90% power to detect a 1-sided significant difference at alpha of 0.05 between LCPT and Tacrolimus twice-daily. Following the finalization of the study protocol the US Food and Drug Administration (FDA) issued guidance to industry on non-inferiority clinical trials, in which FDA specified that the non-inferiority of the investigational drug versus the control should be based on the upper bound of a 2-sided 95% confidence interval (CI) for the difference. To be consistent with this recommendation, the non-inferiority test for this study was based on the upper bound of a 2-sided 95% CI rather than the upper bound of a 1-sided 95% CI as was specified previously in the original study protocol.

A post hoc analysis on black patients was conducted comparing the primary composite efficacy endpoint and the individual efficacy components to further assess efficacy in this population.

***Primary Efficacy Analysis***

The non-inferiority evaluation for the primary efficacy endpoint was based on the 2-sided 95% CIs for the difference (LCPT – tacrolimus twice-daily) in the efficacy failure rates between the treatment groups. If the upper bound of the 95% CI for the difference in efficacy failure rates was less than 9%, then LCPT was to be considered noninferior to tacrolimus twice-daily in efficacy failure rate.

***Primary Safety Analysis***

The primary safety endpoints were the differences between treatment groups at month 12 with respect to AEs and the incidence of predefined potentially clinically significant laboratory measures. All AEs were summarized with no inferential statistics performed. The analysis of the incidence of each predefined potentially clinically significant laboratory measure was based on the corresponding group of at risk patients (patients whose laboratory values at baseline did not meet the predefined abnormal criteria). For each parameter, the number of patients at risk at baseline and the number of new-onset cases during the 12-month treatment period, the percentages of new-onset cases out of the at-risk group were determined. The new-onset risk difference between the treatment groups was estimated, and the 2-sided 95% CI was calculated using an asymptotic approach. Statistical significance regarding the risk difference between the treatment groups was determined using Fisher exact test.

***Secondary Efficacy Analysis***

Statistical tests were performed at the 2-sided 0.05 significance level and the 2-sided 95% CI were constructed. The incidence of efficacy failure within 6 and 12 months after the first dose of study drug was estimated and analyzed using methods similar to those described for the primary efficacy endpoint analysis. The incidences of death or graft failure, acute allograft rejections, and premature discontinuation and the proportion of severity grades of the first episode of BPAR were summarized using frequencies and percentages within each treatment group. The incidence rates were compared between treatment groups using Fisher exact test; premature discontinuation was calculated at 12 months only. The 95% CIs for the difference in incidence were constructed using an asymptotic approach. The 2-sided 95% CIs for the differences in patient and graft survival at 12 months were calculated using Greenwood’s formula.

***Secondary Safety Analysis***

Statistical tests were performed at the 2-sided 0.05 significance level, and the 2-sided 95% CIs were constructed. At each visit, descriptive statistics were summarized by treatment groups. For each post-baseline visit, change from baseline was calculated and summarized using descriptive statistics. The numbers and percentages of patients who 1) had NODM within 6 and 12 months, 2) experienced any opportunistic infection within 12 months, or 3) experienced any malignancy within 12 months were determined and compared between the treatment groups using Fisher exact test. The 95% CI for the difference was constructed using an asymptotic approach.
